# Diffuse glioma molecular profiling with arterial spin labeling and dynamic susceptibility contrast perfusion MRI: A comparative study

**DOI:** 10.1093/noajnl/vdae113

**Published:** 2024-07-05

**Authors:** Yeva Prysiazhniuk, Andres Server, Henning Leske, Øystein Bech-Aase, Eirik Helseth, Roelant Sjouke Eijgelaar, Elies Fuster-García, Petter Brandal, Atle Bjørnerud, Jakub Otáhal, Jan Petr, Wibeke Nordhøy

**Affiliations:** Department of Pathophysiology, Second Faculty of Medicine, Charles University, Prague, The Czech Republic; Section of Neuroradiology, Department of Radiology and Nuclear Medicine, Oslo University Hospital, Oslo, Norway; Department of Pathology, Oslo University Hospital, Oslo, Norway; Department of Physics and Computational Radiology, Division of Radiology and Nuclear Medicine, Oslo University Hospital, Oslo, Norway; Department of Neurosurgery, Oslo University Hospital, Oslo, Norway; Institute of Clinical Medicine, Faculty of Medicine, University of Oslo, Oslo, Norway; Department of Neurosurgery, Amsterdam University Medical Center, Amsterdam, The Netherlands; Biomedical Data Science Laboratory, Instituto Universitario de Tecnologías de la Información y Comunicaciones, Universitat Politècnica de València, València, Spain; Department of Oncology, Oslo University Hospital, Oslo, Norway; Section for Cancer Cytogenetics, Institute for Cancer Genetics and Informatics, Oslo University Hospital, Oslo, Norway; Department of Physics and Computational Radiology, Division of Radiology and Nuclear Medicine, Oslo University Hospital, Oslo, Norway; Center for Lifespan Changes in Brain and Cognition, University of Oslo, Oslo, Norway; Department of Pathophysiology, Second Faculty of Medicine, Charles University, Prague, The Czech Republic; Helmholtz-Zentrum Dresden-Rossendorf, Institute of Radiopharmaceutical Cancer Research, Dresden, Germany; Department of Radiology and Nuclear Medicine, Amsterdam Neuroscience, Amsterdam University Medical Center, Location VUmc, Amsterdam, The Netherlands; Department of Physics and Computational Radiology, Division of Radiology and Nuclear Medicine, Oslo University Hospital, Oslo, Norway

**Keywords:** ASL, DSC, glioma, IDH, pTERT

## Abstract

**Background:**

Evaluation of molecular markers (*IDH*, p*TERT*, 1p/19q codeletion, and *MGMT*) in adult diffuse gliomas is crucial for accurate diagnosis and optimal treatment planning. Dynamic Susceptibility Contrast (DSC) and Arterial Spin Labeling (ASL) perfusion MRI techniques have both shown good performance in classifying molecular markers, however, their performance has not been compared side-by-side.

**Methods:**

Pretreatment MRI data from 90 patients diagnosed with diffuse glioma (54 men/36 female, 53.1 ± 15.5 years, grades 2–4) were retrospectively analyzed. DSC-derived normalized cerebral blood flow/volume (nCBF/nCBV) and ASL-derived nCBF in tumor and perifocal edema were analyzed in patients with available *IDH*-mutation (*n* = 67), p*TERT*-mutation (*n* = 39), 1p/19q codeletion (*n* = 33), and *MGMT* promoter methylation (*n* = 31) status. Cross-validated uni- and multivariate logistic regression models assessed perfusion parameters’ performance in molecular marker detection.

**Results:**

ASL and DSC perfusion parameters in tumor and edema distinguished *IDH*-wildtype (wt) and p*TERT-*wt tumors from mutated ones. Univariate classification performance was comparable for ASL-nCBF and DSC-nCBV in *IDH* (maximum AUROCC 0.82 and 0.83, respectively) and p*TERT* (maximum AUROCC 0.70 and 0.81, respectively) status differentiation. The multivariate approach improved *IDH* (DSC-nCBV AUROCC 0.89) and p*TERT* (ASL-nCBF AUROCC 0.8 and DSC-nCBV AUROCC 0.86) classification. However, ASL and DSC parameters could not differentiate 1p/19q codeletion or *MGMT* promoter methylation status. Positive correlations were found between ASL-nCBF and DSC-nCBV/-nCBF in tumor and edema.

**Conclusions:**

ASL is a viable gadolinium-free replacement for DSC for molecular characterization of adult diffuse gliomas.

**Key Points**

- ASL and DSC both show good efficacy in classifying *IDH* and p*TERT* alterations.
- Edema perfusion is a new potential marker for improved glioma assessment.
- There is a significant correlation between ASL and DSC parameters in tumor and edema.

**Importance of the Study**

We investigated the potential of noninterventional perfusion methods to classify molecular marker status in glioma, analyzing both tumor and peritumoral edema regions. Moreover, we validated the feasibility of noninvasive ASL as an alternative to DSC for adult glioma assessment in the same-population comparison.

Brain tumors are classified according to the World Health Organization (WHO) Classification of Tumors of the Central Nervous System (WHO CNS), in which molecular, genetic, and histological characteristics are incorporated as diagnostic criteria.^1^ Diffuse gliomas are the most common type of malignant brain tumors in adults and represent a significant challenge in diagnosis and treatment since tumors with different molecular characteristics exhibit varying sensitivity to chemo- and radiotherapy.^2,3^ Hence, an accurate diagnosis is crucial for determining the optimal treatment strategy for each patient. Modern neuroimaging modalities, specifically magnetic resonance imaging (MRI), play a critical role in the diagnostic process, treatment planning, and post-therapeutic follow-up of brain tumors.

Current clinical practice in most instances relies on neuropathological evaluation of tumor tissue to give an accurate diagnosis.^4^ However, for tumors in eloquent areas, a biopsy procedure is associated with risks.^5^ Hence, in some cases, a diagnosis is solely based on radiological features without a tissue-based neuropathological examination. In other cases, the extirpated tissue specimen might not be representative of the whole tumor leading to an inconclusive diagnosis. Advanced MRI methods can identify the most malignant parts of a tumor to optimize biopsy sampling in gliomas, reducing the risk of underestimating tumor grade.^6^ Promising MRI sequences were recently reviewed by Glioma MR Imaging 2.0 (GliMR) COST action.^7,8^ In particular, blood flow and volume have been demonstrated to correlate with tumor vascularization and angiogenesis,^9^ essential parameters of pathophysiological tumor subtyping.^10^ In this context, 2 main perfusion methods are used: Dynamic Susceptibility Contrast (DSC) and Arterial Spin Labeling (ASL). DSC relies on the injection of gadolinium-based contrast agents (GBCA),^11^ whereas ASL is a noninvasive alternative without any additional costs.^12^ Even though ASL eliminates both the potential risks of using GBCA^13^ and the discomfort of contrast agent administration,^14^ the level of clinical validation is lower than for DSC.^7^ Diagnostic performance in glioma was previously investigated for DSC as well as for ASL. Both methods have separately shown to be effective for glioma grading,^15,16^ brain tumor classification,^17^ and differentiation of *IDH*-wildtype (wt) and *IDH*-mutant (mut) neoplasms—a key molecular marker in the 2021 edition of WHO CNS classification system.^18–20^

This study aimed to evaluate and directly compare the DSC- and ASL-derived perfusion parameters in their pretreatment glioma characterization performance. We investigated the ability of ASL to classify molecular marker status, including *IDH* and p*TERT* mutation, 1p/19q codeletion, and *MGMT* promoter methylation. The diagnostic potential of DSC and ASL was compared based on perfusion features derived from the tumor and peritumoral edema regions of interest.

## Materials and Methods

### Population

One hundred and six patients with diffuse glioma (54.2 ± 15.2 years, 66 male/40 female), admitted to Oslo University Hospital, 2011–2021, were retrospectively included in the study. All participants provided written informed consent and the local ethics committee approved the study in accordance with the Declaration of Helsinki. The criteria for inclusion were as follows: age ≥ 18 years, confirmed diagnosis of diffuse glioma based on histological examination, no prior cancer treatment, and available imaging data from a presurgical MRI protocol with DSC and ASL acquired within a single session.

All tumors were tested immunohistochemically for the presence or absence of *IDH1* p.R132H mutant protein and retention or loss of nuclear *ATRX* expression. In cases with immunohistochemical loss of *ATRX* expression, the tumors were tested further with *IDH*-sequencing. In a subset of gliomas additional information on the *TERT*-promoter mutation status, *IDH1/2*-mutation status, 1p/19q-codeletion status, and *MGMT* status was available. All tumors with confirmed *IDH* mutation at position p.132 or p.172 were tested for 1p/19q-codeletion status. Gliomas with *IDH1* or *IDH2* mutation and combined 1p/19q-codeletion have been diagnosed as oligodendrogliomas according to the current CNS WHO classification (2021), whereas those without the presence of complete 1p/19q-codeletion were diagnosed as astrocytoma, *IDH*-mutant. Glioblastomas have been diagnosed based on the absence of an *IDH* mutation with either the presence of a hotspot *TERT*-promoter mutation and/or histomorphological features such as vascular proliferation or necrosis. When only *IDH1* p.R132H negativity and retention of *ATRX* expression were available, the diagnosis of glioblastoma was given in cases where vascular proliferation or necrosis was present and where the age of the patient at initial diagnosis was above 54 years. All other cases were diagnosed as diffuse astrocytomas not otherwise specified (NOS).

### Image Acquisition

**Figure 1.**
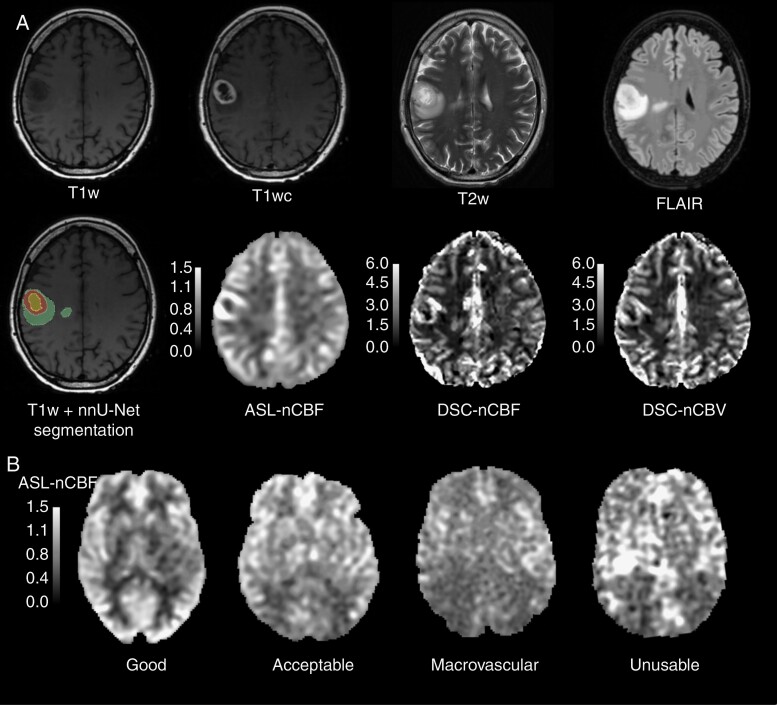
(A) 50-year-old female with glioblastoma, grade 4 (*IDH*-wildtype, 1p/19q noncodeleted, *MGMT*-methylated, and p*TERT*-mutant). Structural segmentation resulting from the nnU-Net model shows necrosis (inner rim, yellow), enhancing tumor (middle rim, red), and edema (outer rim, green). (B) Examples of the ASL quality control assessment. Good—the perfusion signal is well-distributed in gray matter, and there are no visible motion or labeling artifacts; acceptable—minor motion or macrovascular artifacts resulting in regional loss of signal, but the acceptable overall quality, especially around the tumors; macrovascular—prominent macrovascular-signal artifact caused by delayed arterial arrival time; unusable—significant signal distortions, motion artifacts, failed labeling, or too high arrival time.

Imaging data were acquired on 3 different 3 tesla GE (GE Healthcare) MRI platforms: SIGNA™ HDxt in 2011–2016 (*n* = 44), Discovery™ MR750 in 2016–2019 (*n* = 52), SIGNA™ Premier in 2019-2021 (*n* = 18). Details about the MRI system, protocol, and acquisition parameters of structural pre and postcontrast T1-weighted (T1w and T1wc, respectively), T2-weighted (T2w), and T2-weighted fluid-attenuated inversion recovery (FLAIR), ASL, and DSC are in Supplementary Table 1, while representative scans from a single subject are displayed in Figure 1A.

### Image Processing

Structural scans (T1w, T1wc, T2w, and FLAIR) were coregistered using normalized mutual information objective function and resliced to the native T1w space. The respective voxel sizes of T1w were 1.0 × 1.0 × 1.5 mm^3^ (Signa HDxt), 1 mm isotropic (Discovery MR750) and 0.5 mm (interpolated from 1 mm isotropic, on Signa Premier) in Statistical Parametric Mapping 12 (SPM12, version 7765).^21^

Tumor and edema were segmented using the nnU-Net segmentation model^22,23^ on the structural scans (T1w, T1wc, T2w, and FLAIR). The model-generated structural segmentations were validated and adjusted by a board-certified neuroradiologist with 25 years of experience.

ASL images were processed with ExploreASL version 1.10.0^24^ using a simplified single-compartment single-postlabeling delay model.^25^ Brain tissue segmentation using CAT12 (v12.7)^26^ involved masking tumor and necrotic areas (already segmented by the nnU-Net) to mitigate their impact on the normal-appearing tissue segmentation. ASL-CBF maps were normalized to mean CBF in the contralateral hemisphere normal-appearing GM (ASL-nCBF). DSC data were processed in nordicICE (v4.1.2; NordicNeuroLab) with the use of whole-volume arterial input function (AIF), motion and leakage correction, and normalization to automatically detected normal-appearing WM,^27^ yielding normalized cerebral blood volume (CBV) and CBF (DSC-nCBV and DSC-nCBF, respectively). Finally, DSC-nCBF, DSC-nCBV, and ASL-nCBF maps were coregistered and resliced to the structural scans’ space and voxel sizes.

Quality control of ASL-CBF maps was performed by 2 researchers with 10+ and 2 years of experience in ASL (JP and YP). Unclear cases were discussed in a joint session to reach a consensus. ASL scans were classified as “good,” “acceptable,” “macrovascular,” and “unusable” based on the number of macrovascular artifacts and the general image quality (Figure 1B).

### ROI Analysis

Tumor and edema segmentations were used to create the regions of interest (ROI) for quantitative perfusion analysis. For ASL, voxels with a distance less than 5 mm from the tumor mask were excluded from the edema mask to minimize the effect of partial volume effects (PVE) and signal contamination between tissues. Cases with missing edema segmentations or edema volumes smaller than 0.5 cm^3^ were excluded from the edema analysis. Several statistical descriptors (5th percentile, median, 95th percentile, and interquartile range (IQR)) were extracted for edema and tumor ROIs in the normalized perfusion maps ASL-nCBF, DSC-nCBF, and DSC-nCBV. These 4 descriptors, 3 perfusion parameters, and 2 ROIs yielded together 24 parameters.

### Data Analysis

Group differences in the above-mentioned statistical descriptors were assessed in groups stratified according to *IDH* mutation, 1p/19q codeletion, p*TERT* mutation, and *MGMT* methylation using a Cohen’s *d* with Hedge’s correction and a two-tailed Student’s *t*-test with Satterthwaite’s approximation for unequal variances. Additionally, Cohen’s *d* and its confidence interval were estimated with bootstrapping with 1000 iterations. Benjamini-Hochberg procedure was subsequently used to account for multiple comparison bias. The significance level was set to *P* ≤ .05. To investigate the synergistic effect of molecular markers on tumor and edema perfusion, the best-performing ASL and DSC descriptors in tumor and edema were compared between subgroups of markers that had shown significant perfusion differences and between diffuse glioma entities. Additionally, all values were compared between DSC- and ASL-derived parameters using Spearman correlation. Consequently, derived effect sizes were used for the power analysis (alpha = 0.05 and power = 0.8).

Univariate logistic regression models classifying the molecular status were built separately for each of the 24 statistical descriptors. Their performance was assessed with the area under the receiver operating characteristic curve (AUROCC), sensitivity, and specificity. Bootstrapping with 1000 iterations was used to derive the confidence intervals.

Multivariate classification models, using a multivariate logistic regression model with LASSO regularization and 10-fold cross-validation, were implemented to include both edema and tumor perfusion parameters. Every such model was built separately on ASL-nCBF, DSC-nCBV, and DSC-nCBF descriptors. The multivariate approach was applied in the subset where both edema and tumor segmentations were present. Due to the insufficient data on 1p/19q codeletion and *MGMT* methylation status, multivariate classification was only analyzed for *IDH* and p*TERT* alterations.

## Results

**Figure 2.**
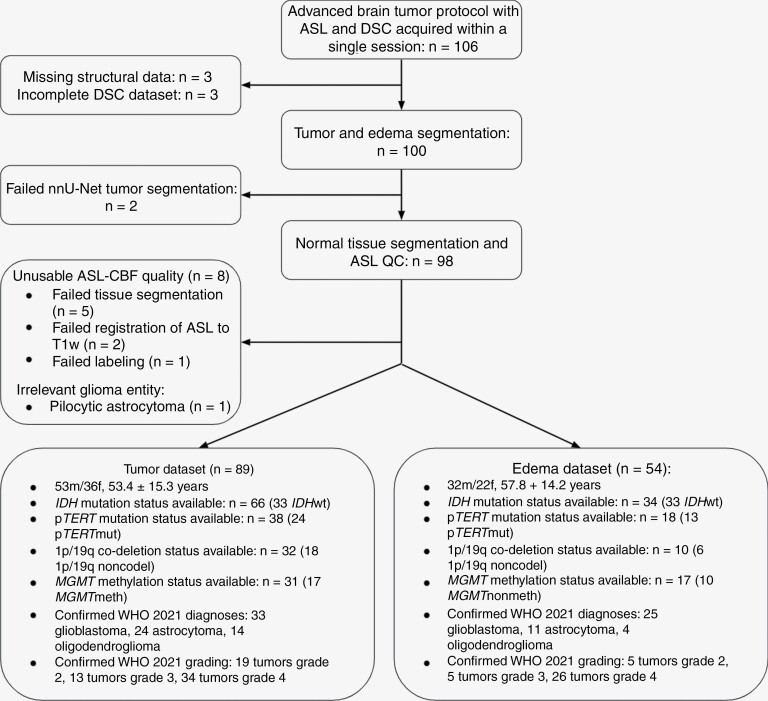
Flowchart of the present study.

The final dataset included 89 patients (53 male, 53.4 ± 15.3 years, grades 2–4) (Figure 2). Overall, *IDH* mutation status was available for 66 subjects, p*TERT* mutation status for 38 subjects, 1p/19q-codeletion status for 32 subjects, and *MGMT* methylation status for 31 subjects. Histopathological status was confirmed for 33 subjects with glioblastoma, 24 subjects with astrocytoma, and 14 subjects with oligodendroglioma. The majority of ASL images were rated as “good” (73.3%), but “acceptable” (13.3%) and “macrovascular” scores (13.3%) were consistently present across platforms (Supplementary Table 2). Eight subjects were excluded because ASL data were rated as “unusable.” Edema segmentation of substantial volume met the inclusion criteria in 54 subjects.

### Perfusion Signal Distribution and Molecular Markers

The analysis of signal distributions revealed significant variations across multiple DSC and ASL perfusion parameters depending on the molecular marker status (Table 1). Specifically, in *IDH-*wt gliomas, significantly higher perfusion values were evident in tumors for multiple ASL-nCBF, DSC-nCBV, and DSC-nCBF descriptors. The largest effect sizes between the *IDH-*wt and IDH-mut gliomas were observed in DSC-nCBV tumor median (Cohen’s *d* = 1.21) and ASL-nCBF tumor 5th percentile and median (Cohen’s *d* = 1.19 and 1.18). In p*TERT* subgroups, multiple ASL- and DSC-related parameters both in tumor and peritumoral edema exhibit significant signal differences between p*TERT*-mut and p*TERT*-wt gliomas. In tumor, only 5th percentile ASL-nCBF shows a large effect size (Cohen’s *d* = 0.81), while in edema, multiple DSC-nCBV (median, 95th percentile, IQR) descriptors show a large effect size (Cohen’s *d* > 0.8). Within 1p/19q—codeletion and *MGMT* methylation subgroups, although no statistical significance is observed, Cohen’s *d* values indicate substantial mean differences in ASL-nCBF values within edema (Supplementary Table 3). DSC-nCBF descriptors have large effect sizes in *IDH* and p*TERT* status differentiation, however, when compared to DSC-nCBV, they show a lower discriminative power (Supplementary Table 4). The retrospective power analysis results of the feasible sample sizes for a reliable comparison of ASL and DSC’s most prominent perfusion parameters in the classification of p*TERT* mutation, 1p/19-codeletion, and MGMT alteration status are presented in Supplementary Table 5.

**Table 1.** Perfusion Signal Distributions in Tumor and Edema of IDH-Mutant/Wildtype and p*TERT*-Mutant/Wildtype Gliomas. *Indicates Significant *P*-values (*P* < .05) after the Benjamini-Hochberg Procedure. Highlighted in Bold are the Significant *P*-values and Cohen’s *d*-Values > 0.8. *n* Indicates the Sample Size

*I* *DH*								
	Tumor				Edema			
Perfusion parameter	*IDH*mut (mean ± SD)	*IDH*wt (mean ± SD)	Cohen’s *d* (95% CI)	*P*-value	*IDH*mut (mean ± SD)	*IDH*wt (mean ± SD)	Cohen’s *d* (95% CI)	*P*-value
	*n* = 33	*n* = 33			*n* = 11	*n* = 23		
ASL-nCBF 5th percentile	0.45 ± 0.14	0.65 ± 0.18	**1.19** (0.6 – 1.71)	**8.3e**–6*	0.41 ± 0.11	0.42 ± 0.16	0.04 (0–0.6)	.89
ASL-nCBF median	0.81 ± 0.28	1.23 ± 0.42	**1.18** (0.69–1.68)	**1.1e**–5*	0.88 ± 0.68	0.68 ± 0.23	0.45 (0–1.52)	.37
ASL-nCBF 95th percentile	1.33 ± 0.75	2.09 ± 0.79	**0.97** (0.32–1.58)	**1.7e**–4*	1.35 ± 1.02	1.29 ± 0.75	0.06 (0–1.18)	.87
ASL-nCBF IQR	0.40 ± 0.39	0.61 ± 0.31	0.58 (0.02–1.24)	**.0208**	0.41 ± 0.49	0.34 ± 0.31	0.18 (0–1.45)	.68
DSC-nCBV 5th percentile	0.36 ± 0.47	0.53 ± 0.86	0.24 (0–0.69)	.32	0.13 ± 0.39	0.16 ± 0.28	0.08 (0–0.95)	.84
DSC-nCBV median	1.50 ± 0.62	3.06 ± 1.69	**1.21** (0.75–1.63)	**1.2e**–5*	1.27 ± 0.44	1.38 ± 0.56	0.21 (0–0.9)	.54
DSC-nCBV 95th percentile	4.40 ± 1.49	7.60 ± 3.59	**1.15** (0.75–1.56)	**2.5e**–5*	4.34 ± 1.07	4.75 ± 1.30	0.33 (0–0.97)	.34
DSC-nCBV IQR	1.52 ± 0.73	2.85 ± 1.45	**1.15** (0.72–1.55)	**2.2e**–5*	1.29 ± 0.40	1.58 ± 0.70	0.46 (0–1.05)	.13

p*TERT*								
	Tumor				Edema			
Perfusion parameter	p*TERT*mut (mean ± SD)	p*TERT*wt (mean ± SD)	Cohen’s *d* (95% CI)	*P*-value	p*TERT*mut (mean ± SD)	p*TERT*wt (mean ± SD)	Cohen’s *d* (95% CI)	*P*-value
	*n* = 24	*n* = 14			*n* = 13	*n* = 5		
ASL-nCBF 5th percentile	0.61 ± 0.19	0.47 ± 0.10	**0.81** (0.21–1.45)	**.0067**	0.45 ± 0.11	0.44 ± 0.11	0.09 (0–1.06)	.85
ASL-nCBF median	1.11 ± 0.38	0.84 ± 0.27	0.76 (0.04–1.38)	**.0156**	0.88 ± 0.63	0.62 ± 0.13	0.45 (0–0.9)	.18
ASL-nCBF 95th percentile	1.88 ± 0.83	1.40 ± 0.73	0.58 (0–1.12)	.0783	1.72 ± 1.24	0.93 ± 0.18	0.70 (0.25–1.11)	**.0433**
ASL-nCBF IQR	0.57 ± 0.43	0.42 ± 0.37	0.34 (0–0.85)	.28	0.25 ± 0.57	0.19 ± 0.04	0.64 (0.33–1.07)	.0597
DSC-nCBV 5th percentile	0.42 ± 0.66	0.38 ± 0.50	0.07 (0–0.69)	.82	0.14 ± 0.22	0.07 ± 0.48	0.22 (0–3.2)	.76
DSC-nCBV median	2.47 ± 1.51	1.61 ± 0.68	0.66 (0.17–1.17)	**.0217**	1.45 ± 0.52	0.85 ± 0.47	**1.13** (0.31–2.14)	**.0457**
DSC-nCBV 95th percentile	6.76 ± 3.70	4.76 ± 1.32	0.64 (0.16–1.06)	**.0223**	5.10 ± 1.30	3.40 ± 1.40	**1.22** (0–2.33)	.0517
DSC-nCBV IQR	2.57 ± 1.58	1.58 ± 0.61	0.74 (0.14–1.14)	**.0099**	1.76 ± 0.74	0.99 ± 0.33	**1.13** (0.39–1.85)	**.0075**

### Subgroup Analysis

*IDH* and p*TERT* markers were chosen for the subgroup analysis with tumor mean and edema 95th percentile of ASL-nCBF and DSC-nCBV as best-performing features (Supplementary Table 6). Despite limited sample sizes, significant differences were observed in tumor and edema between p*TERT*-mut and p*TERT-*wt gliomas within the *IDH*-wt subgroup (*P* = .0081 for tumor median ASL-nCBF and *P* = .0143 for 95th percentile of edema DSC-nCBV). Median tumor perfusion significantly differentiated astrocytomas and glioblastomas, as well as oligodendrogliomas and glioblastomas for both ASL-nCBF and DSC-nCBV (corrected *P*-value < .05) (Supplementary Table 7). Significant perfusion signal differences were observed in both ASL-nCBF and DSC-nCBV median tumor parameters in LGG and HGG subgroups (corrected *P*-value < .05) (Supplementary Table 8).

### Univariate Classification

**Figure 3.**
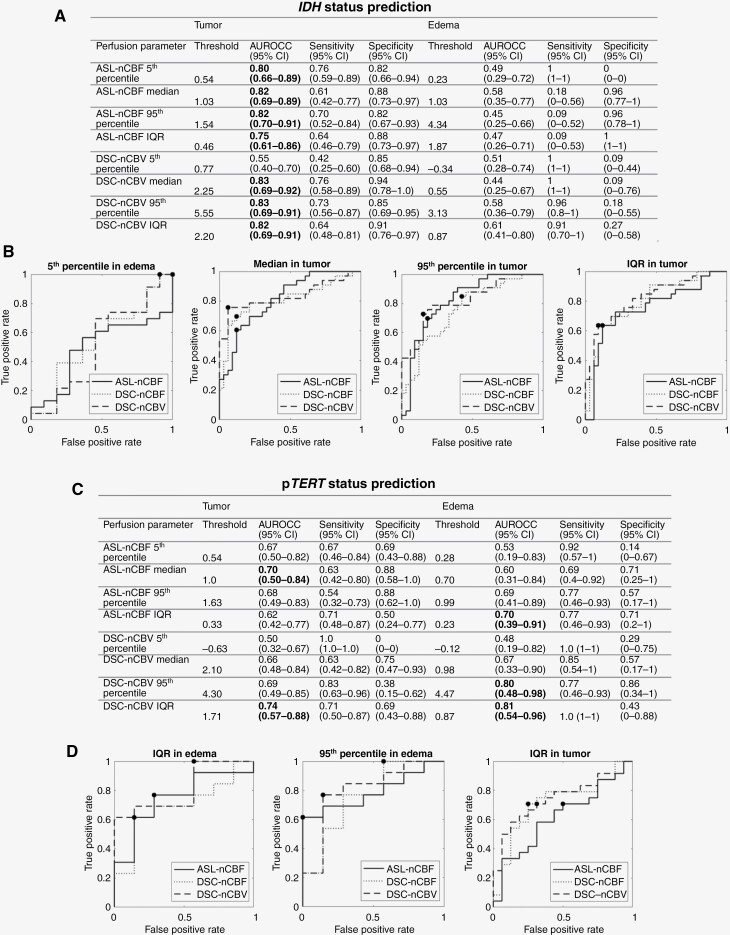
*IDH* and p*TERT* status prediction performance of ASL-nCBF and DSC-nCBV univariate models. (A) *IDH* prediction performance metrics. Highlighted in bold are AUROCC ≥ 0.7. (B) Receiver operating characteristic curves of *IDH*-classifying perfusion descriptors with the highest AUROCC. (C) p*TERT* prediction performance metrics. Highlighted in bold are AUROCC ≥ 0.7. (D) Receiver operating characteristic curves of p*TERT* classifying perfusion descriptors with the highest AUROCC.

Good performance (AUROCC around 0.8) was demonstrated in differentiation of the *IDH* mutation status using a single descriptor of ASL-nCBF or DSC-nCBV (Figure 3A and B), with the highest AUROCC achieved by the median tumor DSC-nCBV (AUROCC = 0.83). Both tumor and edema perfusion parameters showed potential in classifying p*TERT* status (Figure 3C and D). Here, the IQR and 95th percentile of DSC-nCBV within edema demonstrated superior performance, achieving the highest AUROCC (0.81 and 0.8). In the task of classifying the 1p/19q-codeletion status, only ASL-nCBF parameters (IQR and median) within the edema region showed strong performance (AUROCC 0.85 and 0.81, respectively) (Supplementary Table 9). Finally, none of the perfusion parameters exhibited potential in the univariate classification of the *MGMT* methylation status (Supplementary Table 9). Despite some DSC-nCBF parameters showing high *IDH* and p*TERT* status classification performance (AUROCC > 0.73), their efficacy was inferior to DSC-nCBV parameters (Supplementary Table 10).

### Multivariate Classification

**Figure 4.**
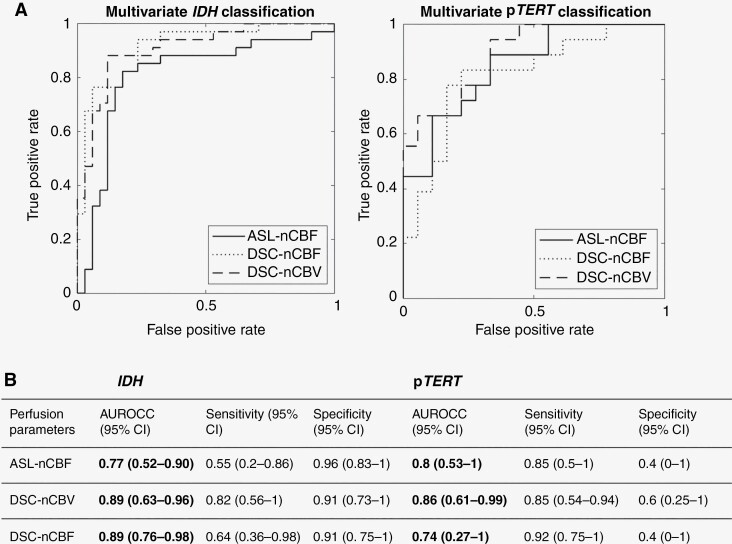
Predictive performance of multivariate logistic regression models built on ASL- and DSC-perfusion parameters. (A) ROC curves of *IDH* and p*TERT* classification models. (B) Performance metrics of *IDH*- and p*TERT*-classification models.

Multivariate models based on ASL- and DSC-derived parameters showed better performance in differentiating *IDH* and p*TERT* mutation status than the respective univariate models (Figure 4). For *IDH* status classification, DSC-nCBF and DSC-nCBV parameters achieved higher AUROCC (0.89 and 0.89, respectively) compared to ASL-nCBF (0.77). For p*TERT* mutation status, ASL-nCBF demonstrated a comparable AUROCC (0.8) to both DSC-nCBF and DSC-nCBV (0.74 and 0.86, respectively).

### Correlation Analysis

**Figure 5.**
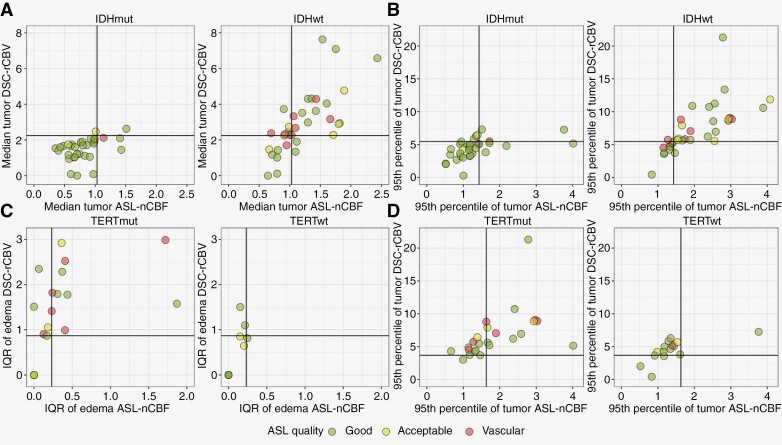
Scatterplots of ASL-nCBF and DSC-nCBV parameters in *IDH*-wildtype/mutant and p*TERT*-wildtype/mutant. TP—true positive, TN—true negative, FP—false positive, FN—false negative. Black horizontal lines indicate thresholds of univariate predictive models. Here, *IDH*-wildtype/mutant and p*TERT*-mutant/wildtype represent positive/negative cases respectively, according to the signal distribution analysis.

Scatterplots of key ASL-nCBF and DSC-nCBV classification features in tumor and edema are displayed in Figure 5. ASL data affected by macrovascular and minor motion artifacts were comparable to the good-quality data in terms of *IDH* and p*TERT* status differentiation. The results of the correlation analysis indicated a stronger correlation between ASL and DSC perfusion parameters in the tumor region compared to the edema (Supplementary Table 11). A significant correlation was observed between ASL-nCBF and DSC-nCBF, as well as ASL-nCBF and DSC-nCBV in all statistical descriptors except the 5th percentile in edema. The correlation between ASL-nCBF and DSC-nCBV was stronger than between ASL-nCBF and DSC-nCBF in the 95th percentile and IQR in both tumor and edema.

## Discussion

In this study, we compared the diagnostic value of ASL- and DSC-derived perfusion parameters in tumor and peritumoral edema for glioma molecular characterization. We found group differences between *IDH* and p*TERT* mutation subgroups of diffuse gliomas in multiple ASL and DSC parameters, while no statistically significant difference was observed between 1p/19q-non/codeleted and *MGMT*-non/methylated subgroups. Single-feature models performed well in classifying tumors by *IDH* and p*TERT* status, with comparable results for ASL and DSC. Lastly, the multivariate approach increased performance in classifying *IDH* and p*TERT* alterations compared to the univariate models and underlined the feasibility of using the noninvasive ASL method as an alternative to the gadolinium-based DSC method in clinical diagnostics of glioma.

In line with existing knowledge on high vascularization and perfusion of *IDH*-wt tumors,^28,29^ our study shows significantly higher tumor perfusion in *IDH*-wt compared with *IDH*-mut gliomas for both ASL and DSC. Group differences are comparable to previous maximum tumor DSC-nCBV^30^ and mean and maximum tumor ASL-CBF^31^ findings. *IDH* mutation showed no effect on peritumoral edema perfusion. Univariate *IDH* status classification revealed comparable performance for ASL and DSC (AUROCC = 0.82 and AUROCC = 0.83, respectively), surpassing values reported by Hosur et al.^18^ (maximum tumor DSC-nCBV AUROCC = 0.66 and maximum tumor ASL-nCBF AUROCC = 0.602). However, they are consistent with the recent meta-analyses on *IDH* status prediction with DSC-nCBV parameters (pooled AUROCC = 0.813,^20^ AUROCC = 0.745–0.911^32^) and previously reported performance metrics for ASL (AUROCC = 0.78).^33^ The multivariate approach improved the DSC performance up to AUROCC = 0.89 by incorporating multiple histogram parameters into the classification model. For ASL, the multivariate model performance is lower compared to the univariate, potentially owing to the smaller dataset, which reduces the model’s robustness. Our investigation complements previous research by directly comparing ASL and DSC in the same population and confirms the comparable performance of both methods in *IDH* status prediction.

In contrast to the *IDH* status, ASL and DSC showed perfusion variations in both the tumor and edema regions with respect to p*TERT* alterations. However, after statistical correction, the signal differences fail to maintain statistical significance. The classification performance of maximum tumor DSC-nCBV (AUROCC = 0.69) and ASL-nCBF (AUROCC = 0.68) is comparable to the mean tumor DSC-nCBV performance previously reported by Zhang et al. (AUROCC = 0.653),^34^ with a sample size of 43. Moreover, perfusion heterogeneity in peritumoral edema provided a better performance (IQR of edema DSC-nCBV, AUROCC = 0.81). Multivariate models showed superior results compared to prior findings with DSC-nCBV (maximum AUROCC = 0.86), but ASL-nCBF also showed good efficacy (maximum AUROCC = 0.80).

Contrary to earlier reports of elevated nCBV in 1p/19q-codeleted gliomas,^20^ our study shows nonsignificant differences, aligning with the results of other investigations.^35^ While maximum tumor DSC-nCBV previously demonstrated high performance in 1p/19q codeletion detection (AUROCC = 0.73),^36^ we did not observe the high performance of ASL or DSC tumor perfusion parameters. However, the promising performance of ASL perfusion in peritumoral edema was observed (maximum AUROCC = 0.85). Given the unclear role of 1p/19q-codeletion in tumor vasculature, our findings prompt further investigation into how it affects peritumoral edema regions. The higher effect sizes and superior performance of ASL compared to DSC perfusion parameters could underscore the significance of microvasculature in 1p/19q-codeleted gliomas.

The impact of *MGMT* promoter methylation on tumor perfusion is debated. Some studies report a significant perfusion signal difference, such as a study by Yoo et al. (maximum tumor ASL-nCBF effect size 0.37)^37^ and a study by Zhang et al. (mean tumor DSC-nCBV effect size 0.67),^34^ while studies by Song et al.^38^ and Fuster-Garcia et al.^39^ revealed no distinction. Our study found no significant perfusion signal difference in *MGMT*-non/methylated gliomas.

The subgroup analysis revealed that, despite limited sample sizes, significantly different perfusion patterns can be observed between p*TERT*-mut and p*TERT*-wt subgroups of *IDH*-wt gliomas and between *IDH*-wt and IDH-mut subgroups of p*TERT*-mut gliomas. This finding has the potential to validate the synergetic effect of molecular markers in glioma diagnosis and prompt future diffuse glioma classification development. The analysis of perfusion differentiation between the entities validated that perfusion in tumor and edema were significant markers to discriminate oligodendrogliomas and astrocytomas from glioblastomas.

The correlation analysis highlights potential similarities and differences between ASL and DSC parameters in tumor and peritumoral edema. Most parameters exhibit a significant positive correlation between the methods, aligning with prior studies in brain tumors.^40,41^ The significant positive correlation in IQR parameters highlights the potential of ASL as an alternative to DSC in advanced tumor perfusion analysis approaches, such as vascular habitats or fractional tumor burden.^42,43^ On the contrary, the correlation between ASL and DSC perfusion parameters is least pronounced in the 5th percentile of edema and tumor perfusion. This observation suggests potential contributions from varying vessel sizes to the perfusion signal. Specifically, the susceptibility-weighted (T2*) DSC perfusion profile predominantly arises from large tortuous vessels, whereas the ASL perfusion profile primarily originates from the microvasculature.^44^ Other potential sources of this disparity are the higher level of noise expected in the 5th percentile and the choice of different reference tissues for ASL and DSC.^25,45^ Furthermore, the correlation coefficients are lower in the peritumoral edema compared to the tumor region, potentially due to vascular compression^46^ and prolonged arterial transit times, especially impacting the single-delay ASL perfusion with insufficient short postlabeling delay (PLD). This issue can be addressed through the multi-delay ASL approach; however, currently, there is no consensus on its need for brain tumor imaging.^47^ Analysis of the impact of ASL data quality on the molecular marker classification shows that the scans affected by macrovascular-signal and minor motion artifacts can be feasibly used for tumor perfusion assessment.

This study has several limitations. The data analyzed in this study were collected over a long period and were affected by the WHO CNS classification changes and 2 system upgrades, which prevented consistent data collection. However, the molecular marker assessment was consistently carried out often prior to the classification revision, which did not bias the dataset. To avoid inconsistencies regarding the classification changes in glioma grading, we rely on the molecular markers analysis. Moreover, the changes in the structural MRI protocols have a negligible impact on the ROI segmentation and analysis. Platform upgrades have impacted the ASL data acquisition since the oldest sub dataset was acquired with shorter PLD, though the number of scans with arterial transit time artifacts was comparable across all platforms. The issue with shorter labeling duration (LD) than recommended in the ASL sequence remains present in the standard GE ASL sequence.^25^ The use of longer LD can potentially improve the prediction performance of ASL-based models due to the increased signal-to-noise ratio. Additionally, it is important to note that this study has implications only for pretreatment glioma diagnostics and cannot be generalized to posttreatment monitoring.

The choice of a multivariate logistic model is justified for its straightforward interpretability and robustness; however, more advanced predictive models can enhance classification efficiency. With our chosen approach, we also support the future use of perfusion parameters in multiparametric predictive models for glioma diagnostics and contribute to the interpretability of perfusion MRI in the field of Neuro-oncology. An important study limitation is the insufficient sample size for conclusive multi-marker analysis. Despite insignificant perfusion parameter differences, high effect sizes and good classification performances suggest the need for larger populations for conclusive outcomes.

## Conclusions

Both ASL and DSC show great potential for noninterventional molecular characterization of adult gliomas. *IDH*-mutant gliomas have a significantly lower ASL and DSC tumor perfusion compared to *IDH*-wildtype, whereas p*TERT*-mutant gliomas show significantly higher ASL and DSC perfusion in tumor and edema compared to p*TERT-*wildtype. 1p/19q-codeletion and *MGMT* methylation did not have a significant impact on the perfusion in tumor and edema. Univariate predictive models based on ASL and DSC perfusion parameters in tumor regions and edema showed comparable good performance when classifying the *IDH* and p*TERT* mutation status. Multivariate logistic regression models showed improved predictive performance when classifying *IDH* and p*TERT* status. The comparable classification performance of ASL-nCBF to DSC-nCBF and DSC-nCBV underlines the feasibility of using ASL as a noninvasive alternative to DSC for perfusion measurement in glioma diagnosis.

## Supplementary Material

vdae113_suppl_Supplementary_Tables_S1-S11

## Data Availability

All data will be made available by the authors upon request from the corresponding author.
