# Supplementary material for: Diffuse glioma molecular profiling with arterial spin labeling and dynamic susceptibility contrast perfusion MRI: A comparative study
Source: Neurooncol Adv. 2024 Jul 5;6(1):vdae113. doi: 10.1093/noajnl/vdae113 (PMC11259011; doi:10.1093/noajnl/vdae113)
Supplement: vdae113_suppl_Supplementary_Tables_S1-S11 [file vdae113_suppl_supplementary_tables_s1-s11.docx]

|  | GE Signa HDxt  8-channel HNS coil | GE Discovery MR750  32-channel HC | GE Signa Premier  48-channel AIR™ HC |
| --- | --- | --- | --- |
| **Structural sequence parameters** | | | |
| T1w  (interpolation, TR/TE/ETL ms, FA°) | 1x1x1.5 mm^3^ 3D FSPGR  (-/6.6/2.3/- ms, 20°) | 1x1x1 mm^3^ 3D FSPGR (BRAVO)  (-/8.2/3.2/- ms, 12°) | 1x1x1 mm^3^ 3D FSE (CUBE)  (zip 2/512 zip, 552/14/22 ms, 12°) |
| T1w post contrast (T1wc)  (TR/TE/ETL ms, FA°) | 1x1.071x5 mm^3^ 2D SE  (660/14/- ms, 90°) | 1x1x1 mm^3^ 3D FSPGR (BRAVO)  (-/8.2/3.2/- ms, 12°) | 1x1x1 mm^3^ 3D FSE (CUBE)  (zip 2/512 zip, 552/14/22 ms, 12°) |
| T2w  (TR/TE/ETL ms, FA°) | 0.5x0.5x5 (gap 1) mm^3^ 2D FSE (Propeller)  (~6000/~84/28 ms, 90°) | 0.5x0.5x4 (gap 1) mm^3^ 2D FSE (Propeller) (~6500/~120/26 ms, 142°) | 0.5x0.5 x 3 (gap 0.3) mm^3^ 2D FSE (Propeller)  (9968/118/26 ms, 142°) |
| T2w FLAIR  (TR/TI/TE/ETL ms, FA°) | 0.625x0.5x5 (gap 1) mm^3^ 2D IR-FS  (9500/2250/120/0 ms, 90°) | 0.5*0.5*0.5 mm^3^ 3D IR-FSE (CUBE) (8000/2068/91/160 ms, 90°)  After Sept. 2018:  1x1x1.2 mm^3^ 3D IR-FSE (T2 prep, CUBE) (7000/1919/90/160 ms, 90°) | 1x1x1.2 mm^3^ 3D IR-FSE (T2 prep, CUBE, HyperSense) (7000/1942/110/160 ms, 90°) |
| **ASL parameters** | | | |
| Readout | 3D PCASL FSE stack-of-spiral | | |
| Acquired resolution | 512 points/8 arms (recon 128 matrix, FOV 24 cm) | | |
| Reconstructed voxel | 3.79 × 3.79 × 3.0 mm^3^ | | |
| TR/TE ms, FA° | 4836 / 10.4 ms, 155° | 5025 / 11.1 ms, 111° | 5122 / 55.9 ms, 111° |
| Scan duration (min:s) | 4:37 | 4:48 | 4:48 |
| LD/PLD (ms) | 1450 / 1525 | 1450 / 2025 | 1450 / 2025 |
| Other | NEX 3, M0 scan included, 4 pulses BS, labeling plane positioned at 2 cm distance from the base of cerebellum approximately at the C2/C3 vertebrae | | |
| **DSC parameters (consistent across platforms)** | | | |
| Readout | Gradient-recalled EPI (2D GRE-EPI) | | |
| Acquired resolution | Matrix 128 × 128, FOV 24 cm, slice thickness 5.0 (gap 1) mm, 26 slices | | |
| TR/TE ms, FA° | 1875/45 ms, 60**°** | | |
| Contrast administration | A dose of 0.1 mmol/kg gadolinium-based contrast agent was given with a power injector at a rate of 5 ml/s with a subsequent 15 ml saline flush | | |
| Dynamic parameters | Temporal resolution 2.0 s, total scan time 84 s, 53 phases, baseline duration 7 s | | |

**Supplementary Table 1**: *MRI acquisition parameters across different MRI platforms. HNS - head neck spine, T1w T1-weighted, T2w - T2-weighted, FSPGR - fast spoiled gradient echo, SE - spin echo, IR-FSE - inversion recovery fast spin echo, FLAIR - fluid-attenuated inversion recovery, PCASL - pseudo-continuous ASL, TR - repetition time, TE - echo time, ETL - echo train length, FA - flip angle, LD - labeling duration, PLD - post labeling delay, M0 - equilibrium magnetization, iso - isotropic, recon - reconstructed, res - resolution, BS - Background suppression, gadolinium-based contrast agent - gadoterate meglumine; Dotarem®, Guerbet, Aulnay-sous-Bois, France, and Clariscan®, GE Healthcare, USA.*

|  | MRI platform | | |
| --- | --- | --- | --- |
| ASL quality | Signa HDxt | Discovery MR750 | Signa Premier |
| Good | 20 | 36 | 9 |
| Acceptable | 5 | 3 | 4 |
| Macrovascular | 3 | 5 | 4 |
| Unusable | 1 | 6 | 1 |

**Supplementary Table 2**: *Quality-control results of ASL for the different platforms*

| **1p/19q** | | | | | | | | |
| --- | --- | --- | --- | --- | --- | --- | --- | --- |
|  | **Tumor** | | | | **Edema** | | | |
| Perfusion parameter | 1p/19q co-del (mean ± SD) | 1p/19q nonco-del  (mean ± SD) | Cohen’s d (95% CI) | p-value | 1p/19q co-del (mean ± SD) | 1p/19q nonco-del  (mean ± SD) | Cohen’s d (95% CI) | p-value |
|  | n = 14 | n = 18 |  |  | n = 4 | n = 6 |  |  |
| ASL-nCBF 5^th^ percentile | 0.47 ± 0.19 | 0.55 ± 0.19 | 0.42 (0-1.10) | 0.25 | 0.44 ± 0.14 | 0.43 ± 0.11 | 0.07 (0-1.67) | 0.91 |
| ASL-nCBF median | 0.84 ± 0.31 | 0.92 ± 0.34 | 0.23 (0-0.9) | 0.51 | 1.32 ± 1.05 | 0.66 ± 0.12 | **0.91** (0-2.5) | 0.30 |
| ASL-nCBF 95^th^ percentile | 1.41 ± 0.85 | 1.46 ± 0.63 | 0.07 (0-0.8) | 0.86 | 2.01 ± 1.57 | 1.09 ± 0.21 | **0.85** (0-2.41) | 0.32 |
| ASL-nCBF IQR | 0.43 ± 0.48 | 0.39 ± 0.22 | 0.12 (0-1.1) | 0.76 | 0.73 ± 0.76 | 0.26 ± 0.08 | **0.91** (0-2.23) | 0.30 |
| DSC-nCBV 5^th^ percentile | 0.37 ± 0.44 | 0.29 ± 0.57 | 0.17 (0-0.91) | 0.62 | 0.25 ± 0.37 | 0.05 ± 0.40 | 0.45 (0-1.73) | 0.46 |
| DSC-nCBV median | 1.57 ± 0.58 | 1.68 ± 1.08 | 0.1 (0-0.79) | 0.75 | 1.52 ± 0.11 | 1.23 ± 0.61 | 0.53 (0-2.19) | 0.31 |
| DSC-nCBV 95^th^ percentile | 4.28 ± 1.46 | 5.04 ± 1.71 | 0.46 (0-1.14) | 0.18 | 4.58 ± 0.32 | 4.71 ± 1.39 | 0.1 (0-2.16) | 0.84 |
| DSC-nCBV IQR | 1.46 ± 0.55 | 1.86 ± 0.83 | 0.53 (0-1.15) | 0.12 | 1.51 ± 0.28 | 1.49 ± 0.54 | 0.03 (0-1.57) | 0.95 |
| ***MGMT*** | | | | | | | | |
|  | **Tumor** | | | | **Edema** | | | |
| Perfusion parameter | *MGMT*meth  (mean ± SD) | *MGMT* nonmeth  (mean ± SD) | Cohen’s d (95% CI) | p-value | *MGMT*meth  (mean ± SD) | *MGMT* nonmeth  (mean ± SD) | Cohen’s d (95% CI) | p-value |
|  | n = 17 | n = 14 |  |  | n = 7 | n = 10 |  |  |
| ASL-nCBF 5^th^ percentile | 0.55 ± 0.22 | 0.56 ± 0.13 | 0.04 (0-0.8) | 0.92 | 0.42 ± 0.11 | 0.46 ± 0.11 | 0.31 (0-1.48) | 0.52 |
| ASL-nCBF median | 0.97 ± 0.38 | 1.01 ± 0.42 | 0.1 (0-0.83) | 0.78 | 1.00 ± 0.85 | 0.69 ± 0.18 | 0.54 (0-2.32) | 0.37 |
| ASL-nCBF 95^th^ percentile | 1.62 ± 0.80 | 1.70 ± 0.85 | 0.09 (0-0.74) | 0.80 | 1.97 ± 1.66 | 1.18 ± 0.44 | 0.67 (0-2.32) | 0.26 |
| ASL-nCBF IQR | 0.48 ± 0.43 | 0.53 ± 0.38 | 0.12 (0-0.88) | 0.73 | 0.70 ± 0.76 | 0.26 ± 0.10 | **0.86** (0-2.44) | 0.18 |
| DSC-nCBV 5^th^ percentile | 0.26 ± 0.52 | 0.51 ± 0.74 | 0.38 (0-1.15) | 0.31 | 0.22 ± 0.26 | 0.03 ± 0.31 | 0.62 (0-1.53) | 0.19 |
| DSC-nCBV median | 2.0 ± 0.93 | 2.29 ± 1.92 | 0.19 (0-0.95) | 0.62 | 1.35 ± 0.55 | 1.18 ± 0.58 | 0.29 (0-1.31) | 0.54 |
| DSC-nCBV 95^th^ percentile | 5.64 ± 1.85 | 6.74 ± 4.80 | 0.31 (0-1.01) | 0.43 | 4.64 ± 1.70 | 4.51 ± 1.48 | 0.08 (0-1.22) | 0.87 |
| DSC-nCBV IQR | 2.16 ± 1.13 | 2.43 ± 1.87 | 0.18 (0-0.94) | 0.64 | 1.53 ± 0.79 | 1.49 ± 0.73 | 0.05 (0-1.14) | 0.92 |
|  |  |  |  |  |  |  |  |  |

**Supplementary Table 3**. *Perfusion signal distributions in tumor and edema of 1p/19q non/co-deleted and MGMT-non/methylated gliomas. Highlighted in bold are Cohen’s d-values >0.8. n indicates the sample size.*

| ***IDH*** | | | | | | | | |
| --- | --- | --- | --- | --- | --- | --- | --- | --- |
|  | Tumor | | | | Edema | | | |
| Perfusion parameter | *IDH*mut (mean ± SD) | *IDH*wt  (mean ± SD) | Cohen’s d (95% CI) | p-value | *IDH*mut (mean ± SD) | *IDH*wt (mean ± SD) | Cohen’s d (95% CI) | p-value |
|  | n = 33 | n = 33 |  |  | n = 11 | n = 23 |  |  |
| DSC-nCBF 5^th^ percentile | 0.24 ± 0.39 | 0.41 ± 0.73 | 0.29  (0-0.76) | 0.24 | 0.07 ± 0.3 | 0.10 ± 0.25 | 0.13  (0-0.94) | 0.75 |
| DSC-nCBF median | 1.42 ± 0.70 | 2.74 ± 1.52 | **1.1** (0.68-1.52) | **4.3e-5*** | 1.25 ± 0.48 | 1.34 ± 0.62 | 0.14  (0-0.81) | 0.67 |
| DSC-nCBF 95^th^ percentile | 4.60 ± 2.04 | 7.39 ± 3.87 | **0.89** (0.48-1.23) | **6.1e-4*** | 4.35 ± 1.49 | 5.15 ± 2.22 | 0.38  (0-0.9) | 0.23 |
| DSC-nCBF IQR | 1.59 ± 0.83 | 2.83 ± 1.50 | **1.0 (0.5-1.4)** | **1.4e-4*** | 1.38 ± 0.541 | 1.74 ± 0.92 | 0.43 (0-0.91) | 0.16 |
| **p*TERT*** | | | | | | | | |
|  | Tumor | | | | Edema | | | |
|  | p*TERT*mut (mean ± SD) | p*TERT*wt (mean ± SD) | Cohen’s d (95% CI) | p-value | p*TERT*mut (mean ± SD) | p*TERT*wt (mean ± SD) | Cohen’s d (95% CI) | p-value |
|  | n = 24 | n = 14 |  |  | n = 13 | n = 5 |  |  |
| DSC-nCBF 5^th^ percentile | 0.30 ± 0.57 | 0.23 ± 0.36 | 0.13  (0-0.76) | 0.66 | 0.09 ± 0.17 | -0.01 ± 0.37 | 0.37 (0-2.44) | 0.61 |
| DSC-nCBF median | 2.28 ± 1.43 | 1.55 ± 0.86 | 0.57  (0.03-1.07) | 0.057 | 1.39 ± 0.57 | 0.79 ± 0.46 | **1.06** (0.29-2.08) | **0.0443** |
| DSC-nCBF 95^th^ percentile | 6.80 ± 4.13 | 5.24 ± 2.34 | 0.42  (0-0.84) | 0.148 | 5.56 ± 2.69 | 3.24 ± 1.19 | **0.92** (0-1.46) | **0.0228** |
| DSC-nCBF IQR | 2.60 ± 1.69 | 1.77 ± 0.93 | 0.55 (0-0.99) | 0.056 | 1.88 ± 1.07 | 1.01 ± 0.36 | **0.88** (0.28-1.4) | **0.0202** |
| **1p/19q** | | | | | | | | |
|  | Tumor | | | | Edema | | | |
|  | 1p/19q codel (mean ± SD) | 1p/19q noncodel (mean ± SD) | Cohen’s d (95% CI) | p-value | 1p/19q codel (mean ± SD) | 1p/19q noncodel (mean ± SD) | Cohen’s d (95% CI) | p-value |
|  | n = 14 | n = 14 |  |  | n = 4 | n = 6 |  |  |
| DSC-nCBF 5^th^ percentile | 0.28 ± 0.42 | 0.19 ± 0.51 | 0.18 (0-0.92) | 0.60 | 0.16 ± 0.29 | -0.01 ± 0.3 | 0.52 (0-1.83) | 0.40 |
| DSC-nCBF median | 1.49 ± 0.68 | 1.59 ± 1.05 | 0.1 (0-0.74) | 0.76 | 1.5 ± 0.16 | 1.22 ± 0.59 | 0.54 (0-1.6) | 0.30 |
| DSC-nCBF 95^th^ percentile | 4.32 ± 1.66 | 5.21 ± 2.07 | 0.45 (0-1.1) | 0.19 | 4.72 ± 0.49 | 4.59 ± 2.03 | 0.07 (0-1.23) | 0.89 |
| DSC-nCBF IQR | 1.49 ± 0.59 | 1.83 ± 0.77 | 0.47 (0-1.14) | 0.17 | 1.61 ± 0.36 | 1.51 ± 0.68 | 0.15 (0-1.64) | 0.77 |
| ***MGMT*** | | | | | | | | |
|  | Tumor | | | | Edema | | | |
|  | *MGMT*meth (mean ± SD) | *MGMT* nonmeth (mean ± SD) | Cohen’s d (95% CI) | p-value | *MGMT*meth (mean ± SD) | *MGMT* nonmeth (mean ± SD) | Cohen’s d (95% CI) | p-value |
|  | n = 17 | n = 14 |  |  | n = 7 | n = 10 |  |  |
| DSC-nCBF 5^th^ percentile | 0.19 ± 0.46 | 0.33 ± 0.6 | 0.25 (0-0.97) | 0.50 | 0.14 ± 0.20 | -0.02 ± 0.24 | 0.7 (0-1.61) | 0.15 |
| DSC-nCBF median | 1.82 ± 0.8 | 2.15 ± 1.89 | 0.23 (0-0.99) | 0.55 | 1.28 ± 0.56 | 1.13 ± 0.65 | 0.23 (0-1.32) | 0.62 |
| DSC-nCBF 95^th^ percentile | 5.65 ± 1.37 | 6.99 ± 5.59 | 0.34 (0-1.27) | 0.40 | 4.63 ± 1.43 | 5.07 ± 3.31 | 0.15 (0-0.96) | 0.72 |
| DSC-nCBF IQR | 2.06 ± 0.73 | 2.61 ± 2.24 | 0.33 (0-1.1) | 0.40 | 1.52 ± 0.63 | 1.69 ± 1.26 | 0.16 (0-0.94) | 0.71 |

**Supplementary Table 4**. *DSC-nCBF distributions in tumor and edema of IDH-mutant/wildtype, pTERT-mutant/wildtype, 1p19q-non/codeleted, and MGMT-non/methylated gliomas. * indicates significant p-values (p<0.05) after the Benjamini-Hochberg procedure. Highlighted in bold are the significant p-values and Cohen’s d-values >0.8.*

| Molecular marker | Perfusion parameter of interest | Feasible sample size of subgroup ASL-nCBF / DSC-nCBV |
| --- | --- | --- |
| p*TERT* | Median tumor | 28 / 37 |
| p*TERT* | 95^th^ percentile edema | 33 / 11 |
| 1p/19q | Median edema | 19 / 55 |
| MGMT | Median tumor | 1570 / 435 |

**Supplementary Table 5**. *Post-hoc power analysis results for the promising perfusion parameters in pTERT mutation, 1p/19q-codeletion, and MGMT alteration status classification*.

| **p*TERT* differentiation within *IDH*wt subgroup** | | | | |
| --- | --- | --- | --- | --- |
| Perfusion parameter | *IDH*wt p*TERT*wt (mean ± SD) | *IDH*wt p*TERT*mut  (mean ± SD) | Cohen’s d (95% CI) | p-value |
|  | n = 4 | n = 17 |  |  |
| Tumor median ASL-nCBF | 0.86 ± 0.13 | 1.19 ± 0.35 | **0.95** (0.35-1.56) | **0.0081** |
| Tumor median DSC-nCBV | 1.76 ± 0.69 | 2.82 ± 1.65 | 0.66 (0-1.22) | 0.067 |
|  | n = 2 | n = 11 |  |  |
| Edema 95^th^ percentile ASL-nCBF | 0.86 ± 0.1 | 1.49 ± 1.05 | 0.6 (0-0.89) | 0.075 |
| Edema 95^th^ percentile DSC-nCBV | 2.86 ± 0.55 | 5.16 ± 1.4 | **1.59** (0.82-2.64) | **0.0143** |
| **p*TERT* differentiation within *IDH*mut subgroup** | | | | |
| Perfusion parameter | *IDH*mut p*TERT*wt (mean ± SD) | *IDH*mut p*TERT*mut (mean ± SD) | Cohen’s d (95% CI) | p-value |
|  | n = 10 | n = 7 |  |  |
| Tumor median ASL-nCBF | 0.83 ± 0.31 | 0.91 ± 0.41 | 0.21 (0-1.39) | 0.68 |
| Tumor median DSC-nCBV | 1.55 ± 0.71 | 1.63 ± 0.51 | 0.11 (0-0.95) | 0.81 |
|  | n = 3 | n = 2 |  |  |
| Edema 95^th^ percentile ASL-nCBF | 0.98 ± 0.23 | 2.95 ± 1.96 | **1.24** (0.55-8.2) | 0.39 |
| Edema 95^th^ percentile DSC-nCBV | 3.76 ± 1.81 | 4.74 ± 0.38 | 0.47 (0-1) | 0.45 |
| ***IDH* differentiation within p*TERT*wt subgroup** | | | | |
| Perfusion parameter | p*TERT*wt *IDH*wt (mean ± SD) | p*TERT*wt *IDH*mut (mean ± SD) | Cohen’s d (95% CI) | p-value |
|  | n = 4 | n = 10 |  |  |
| Tumor median ASL-nCBF | 0.86 ± 0.13 | 0.83 ± 0.31 | 0.12 (0-1.18) | 0.76 |
| Tumor median DSC-nCBV | 1.76 ± 0.69 | 1.55 ± 0.71 | 0.28 (0-1.65) | 0.63 |
|  | n = 2 | n = 3 |  |  |
| Edema 95^th^ percentile ASL-nCBF | 0.86 ± 0.1 | 0.98 ± 0.23 | 0.45 (0-4.04) | 0.48 |
| Edema 95^th^ percentile DSC-nCBV | 2.86 ± 0.55 | 3.76 ± 1.81 | 0.43 (0-1) | 0.49 |
| ***IDH* differentiation within p*TERT*mut subgroup** | | | | |
| Perfusion parameter | p*TERT*mut *IDH*wt (mean ± SD) | p*TERT*mut *IDH*mut (mean ± SD) | Cohen’s d (95% CI) | p-value |
|  | n = 17 | n = 7 |  |  |
| Tumor median ASL-nCBF | 1.19 ± 0.35 | 0.91 ± 0.41 | 0.74 (0-1.64) | 0.14 |
| Tumor median DSC-nCBV | 2.82 ± 1.65 | 1.63 ± 0.51 | **0.81** (0.15-1.35) | **0.0137** |
|  | n = 11 | n = 2 |  |  |
| Edema 95^th^ percentile ASL-nCBF | 1.49 ± 1.05 | 2.95 ± 1.96 | **1.17** (0-6.84) | 0.48 |
| Edema 95^th^ percentile DSC-nCBV | 5.16 ± 1.4 | 4.74 ± 0.38 | 0.3 (0-1.02) | 0.42 |

**Supplementary Table 6**. *Perfusion signal distributions within IDH and pTERT subgroups for the best-performing statistical descriptors in tumor and edema. Highlighted in bold are the significant p-values and Cohen’s d-values >0.8. n indicates the sample size.*

| **Astrocytoma and oligodendroglioma differentiation** | | | | |
| --- | --- | --- | --- | --- |
| Perfusion parameter | Astrocytoma (mean ± SD) | Oligodendroglioma (mean ± SD) | Cohen’s d (95% CI) | p-value |
|  | n = 24 | n = 14 |  |  |
| Tumor median ASL-nCBF | 0.93 ± 0.4 | 0.84 ± 0.31 | 0.22 (0-0.81) | 0.48 |
| Tumor median DSC-nCBV | 1.87 ± 1.18 | 1.57 ± 0.58 | 0.29 (0-0.79) | 0.31 |
|  | n = 11 | n = 4 |  |  |
| Edema 95^th^ percentile ASL-nCBF | 1.07 ± 0.28 | 2.01 ± 1.57 | **1.11** (0-3.47) | 0.32 |
| Edema 95^th^ percentile DSC-nCBV | 4.34 ± 1.08 | 4.58 ± 0.32 | 0.24 (0-0.93) | 0.51 |
| **Astrocytoma and glioblastoma differentiation** | | | | |
| Perfusion parameter | Astrocytoma (mean ± SD) | Glioblastoma (mean ± SD) | Cohen’s d (95% CI) | p-value |
|  | n = 24 | n = 33 |  |  |
| Tumor median ASL-nCBF | 0.93 ± 0.4 | 1.22 ± 0.41 | 0.71 (0.09-1.23) | **0.0098*** |
| Tumor median DSC-nCBV | 1.87 ± 1.18 | 3.01 ± 1.69 | 0.75 (0.18-1.2) | **0.0041*** |
|  | n = 11 | n = 25 |  |  |
| Edema 95^th^ percentile ASL-nCBF | 1.07 ± 0.28 | 1.21 ± 0.73 | 0.21 (0-0.69) | 0.42 |
| Edema 95^th^ percentile DSC-nCBV | 4.34 ± 1.08 | 4.92 ± 1.4' | 0.43 (0-1.13) | 0.19 |
| **Oligodendroglioma and glioblastoma differentiation** | | | | |
| Perfusion parameter | Oligodendroglioma (mean ± SD) | Glioblastoma (mean ± SD) | Cohen’s d (95% CI) | p-value |
|  | n = 14 | n = 33 |  |  |
| Tumor median ASL-nCBF | 0.84 ± 0.31 | 1.22 ± 0.41 | **0.96** (0.3-1.47) | **0.0016*** |
| Tumor median DSC-nCBV | 1.57 ± 0.58 | 3.01 ± 1.69 | **0.97** (0.53-1.33) | **9.07e-5*** |
|  | n = 4 | n = 25 |  |  |
| Edema 95^th^ percentile ASL-nCBF | 2.01 ± 1.57 | 1.21 ± 0.73 | **0.9** (0-4.36) | 0.39 |
| Edema 95^th^ percentile DSC-nCBV | 4.58 ± 0.32 | 4.92 ± 1.4' | 0.25 (0-0.74) | 0.31 |

**Supplementary Table 7**. *Perfusion signal distributions in diffuse glioma entities. * indicates significant p-values (p<0.05) after the Benjamini-Hochberg procedure. Highlighted in bold are the significant p-values and Cohen’s d-values >0.8. n indicates the sample size.*

| **LGG and HGG differentiation** | | | | |
| --- | --- | --- | --- | --- |
|  | Tumor | | | |
| Perfusion parameter | LGG (mean ± SD) n=19 | HGG (mean ± SD) n=47 | Cohen’s d (95% CI) | p-value |
| Tumor median ASL-nCBF | 0.82 ± 0.29 | 1.13 ± 0.43 | 0.77 (0.3-1.16) | **0.0011*** |
| Tumor median DSC-nCBV | 1.56 ± 0.54 | 2.62 ± 1.63 | 0.73 (0.41-1.02) | **1.1e-4*** |
|  | Edema | | | |
|  | LGG (mean ± SD) n=5 | HGG (mean ± SD) n=31 | Cohen’s d (95% CI) | p-value |
| Edema 95^th^ percentile ASL-nCBF | 1.78 ± 1.46 | 1.18 ± 0.63 | 0.77 (0-4.12) | 0.42 |
| Edema 95^th^ percentile DSC-nCBV | 4.66 ± 0.33 | 4.73 ± 1.34 | 0.05 (0-0.47) | 0.78 |

**Supplementary Table 8**. *Perfusion signal distributions in low-grade (LGG) and high-grade (HGG) gliomas. * indicates significant p-values (p<0.05) after the Benjamini-Hochberg procedure. Highlighted in bold are the significant p-values. n indicates the sample size.*

| **1p/19q** | | | | | | | | |
| --- | --- | --- | --- | --- | --- | --- | --- | --- |
|  | Tumor | | | | Edema | | | |
| Perfusion parameter | Threshold | AUROCC  (95% CI) | Sensitivity (95% CI) | Specificity  (95% CI) | Threshold | AUROCC  (95% CI) | Sensitivity  (95% CI) | Specificity  (95% CI) |
| ASL-nCBF 5^th^ percentile | 0.87 | 0.58  (0.38-0.75) | 0.11  (0-0.35) | 1.0  (1.0-1.0) | 0.61 | 0.46  (0.06-0.88) | 0  (0-0) | 1.0  (1.0-1.0) |
| ASL-nCBF median | 1.82 | 0.49  (0.31-0.69) | 0.06  (0-0.28) | 1.0  (1.0-1.0) | 1.03 | **0.81**  **(0.18-1)** | 0.50  (0-1.0) | 1.0  (1.0-1.0) |
| ASL-nCBF 95^th^ percentile | 1.18 | 0.49  (0.31-0.67) | 0.72  (0.47-0.91) | 0.43  (0.22-0.65) | 4.34 | 0.73  (0.24-0.97) | 0.25  (0-1.0) | 1.0  (1.0-1.0) |
| ASL-nCBF IQR | 2.03 | 0.39  (0.21-0.60) | 0.07  (0-0.44) | 1.0  (1.0-1.0) | 0.44 | **0.85**  **(0.44-1)** | 0.50  (0-1.0) | 1.0  (1.0-1.0) |
| DSC-nCBV 5^th^ percentile | 2.01 | 0.54  (0.34-0.72) | 0 (0-0) | 1.0  (1.0-1.0) | 0.73 | 0.69  (0.24-0.98) | 0.25  (0-1.0) | 1.0  (1.0-1.0) |
| DSC-nCBV median | 2.95 | 0.35  (0.19-0.54) | 0.17  (0.05-0.40) | 0.95  (0.76-1.0) | 1.41 | **0.77**  **(0.46-0.93)** | 1.0  (1.0-1.0) | 0.77  (0.43-0.93) |
| DSC-nCBV 95^th^ percentile | 5.23 | 0.47  (0.29-0.67) | 0.50  (0.26-0.71) | 0.67  (0.44-0.84) | 5.58 | 0.61  (0.23-0.88) | 0.50  (0-1.0) | 0.82  (0.50-1.0) |
| DSC-nCBV IQR | 2.16 | 0.48  (0.30-0.68) | 0.39  (0.18-0.63) | 0.81  (0.58-0.94) | 2.92 | 0.58  (0.28-0.82) | 0  (0-0) | 1.0  (1.0-1.0) |
| ***MGMT*** | | | | | | | | |
|  | Tumor | | | | Edema | | | |
|  | Threshold | AUROCC  (95% CI) | Sensitivity  (95% CI) | Specificity  (95% CI) | Threshold | AUROCC (95% CI) | Sensitivity (95% CI) | Specificity (95% CI) |
| ASL-nCBF 5^th^ percentile | 0.43 | 0.56  (0.37-0.75) | 0.93  (0.57-1) | 0.40  (0.19-0.62) | 0.44 | 0.63  (0.33-0.85) | 0.7  (0.32-0.92) | 0.63  (0.20-1.0) |
| ASL-nCBF median | 1.54 | 0.49  (0.28-0.70) | 0.21  (0.06-0.53) | 0.95  (0.74-1.0) | 1.03 | 0.52  (0.17-0.86) | 0.29  (0-0.75) | 1.0  (1.0-1.0) |
| ASL-nCBF 95^th^ percentile | 2.78 | 0.46  (0.27-0.68) | 0.29  (0.08-0.56) | 0.90  (0.71-1.0) | 4.34 | 0.55  (0.21-0.83) | 0.29  (0-0.79) | 1.0  (1.0-1.0) |
| ASL-nCBF IQR | 1.06 | 0.47  (0.27-0.70) | 0.29  (0.08-0.55) | 0.9  (0.67-1.0) | 0.44 | 0.61  (0.24-0.88) | 0.43  (0-0.80) | 1.0  (1.0-1.0) |
| DSC-nCBV 5^th^ percentile | 1.38 | 0.58  (0.35-0.77) | 0.14  (0-0.44) | 1.0  (1.0-1.0) | 0.35 | 0.69  (0.31-0.92) | 0.57  (0.20-1.0) | 0.91  (0.56-1.0) |
| DSC-nCBV median | 7.63 | 0.49  (0.29-0.72) | 0.07  (0-0.37) | 1.0  (1.0-1.0) | 2.42 | 0.53  (0.26-0.83) | 0.14  (0-0.67) | 1.0  (1.0-1.0) |
| DSC-nCBV 95^th^ percentile | 8.80 | 0.48  (0.26-0.71) | 0.36  (0.11-0.64) | 0.95  (0.78-1.0) | 7.22 | 0.51  (0.21-0.8) | 0.14  (0-0.63) | 1.0  (1.0-1.0) |
| DSC-nCBV IQR | 7.88 | 0.47  (0.26-0.68) | 0.07  (0-0.36) | 1.0  (1.0-1.0) | 2.99 | 0.48  (0.17-0.78) | 0.14  (0-0.95) | 1.0  (1.0-1.0) |

**Supplementary Table 9**. *1p19q co-deletion and MGMT methylation status prediction performance of univariate ASL-nCBF and DSC-nCBV models.*

|  |  |  |  |  |  |  |  |  |
| --- | --- | --- | --- | --- | --- | --- | --- | --- |
| Perfusion parameter | Threshold | AUROCC  (95% CI) | Sensitivity  (95% CI) | Specificity  (95% CI) | Threshold | AUROCC  (95% CI) | Sensitivity  (95% CI) | Specificity  (95% CI) |
| ***IDH*** | | | | | | | | |
| DSC-nCBF 5^th^ percentile | 0.74 | 0.55  (0.41-0.69) | 0.33  (0.18-0.52) | 0.94  (0.8-1.0) | -0.39 | 0.55  (0.28-0.74) | 1.0  (1.0-1) | 0.09  (0-0.5) |
| DSC-nCBF median | 2.08 | **0.81**  **(0.68-0.90)** | 0.70  (0.53-0.84) | 0.88  (0.72-0.97) | 0.29 | 0.45  (0.26-0.68) | 1.0  (1.0-1.0) | 0.09  (0-0.44) |
| DSC-nCBF 95^th^ percentile | 4.89 | **0.76**  **(0.62-0.85)** | 0.85  (0.69-0.95) | 0.58  (0.41-0.75) | 2.83 | 0.60  (0.37-0.8) | 1.0  (1.0-1.0) | 0.09  (0-0.46) |
| DSC-nCBF IQR | 2.15 | **0.80**  **(0.67-0.89)** | 0.64  (0.46-0.80) | 0.88  (0.73-0.97) | 0.79 | 0.59  (0.39-0.78) | 1.0  (1.0-1.0) | 0.09  (0-0.5) |
| **p*TERT*** | | | | | | | | |
| DSC-nCBF 5^th^ percentile | -0.49 | 0.49  (0.31-0.67) | 1.0  (1.0-1.0) | 0  (0-0) | -0.12 | 0.47  (0.12-0.81) | 1.0  (1-1) | 0.29  (0-0.75) |
| DSC-nCBF median | 1.17 | 0.63  (0.43-0.79) | 0.88  (0.69-0.96) | 0.31  (0.11-0.58) | 1.02 | 0.66  (0.32-0.9) | 0.77  (0.46-0.94) | 0.57  (0.03-0.88) |
| DSC-nCBF 95^th^ percentile | 4.29 | 0.61  (0.40-0.80) | 0.88  (0.69-1.0) | 0.44  (0.20-0.71) | 3.18 | **0.76**  **(0.44-0.95)** | 1.0  (1-1) | 0.43  (0.13-1) |
| DSC-nCBF IQR | 1.93 | **0.72**  **(0.53-0.87)** | 0.71  (0.50-0.87) | 0.75  (0.50-0.93) | 1.60 | 0.69  (0.4-0.9) | 0.62  (0.33-0.87) | 0.86  (0.33-1) |
| **1p/19q** | | | | | | | | |
| DSC-nCBF 5^th^ percentile | 1.61 | 0.55  (0.36-0.72) | 0  (0-0) | 1.0  (1.0-1.0) | 0.55 | 0.69  (0.31-0.96) | 0.25  (0-1) | 1.0  (1-1) |
| DSC-nCBF median | 3.0 | 0.39  (0.22-0.60) | 0.17  (0-0.41) | 0.91  (0.68-1.0) | 2.19 | 0.67  (0.36-0.9) | 0  (0-0) | 1.0  (1-1) |
| DSC-nCBF 95^th^ percentile | 6.86 | 0.45  (0.27-0.65) | 0.33  (0.13-0.57) | 0.86  (0.65-0.96) | 13.51 | 0.52  (0.24-0.76) | 0  (0-0) | 1.0  (1-1) |
| DSC-nCBF IQR | 2.27 | 0.47  (0.28-0.65) | 0.33  (0.13-0.61) | 0.76  (0.55-0.91) | 4.58 | 0.60  (0.33-0.87) | 0  (0-0) | 1  (1-1) |
| ***MGMT*** | | | | | | | | |
| DSC-nCBF 5^th^ percentile | 1.29 | 0.52  (0.3-0.72) | 0.14  (0-0.43) | 1.0  (1-1) | 0.37 | 0.68  (0.33-0.89) | 0.29  (0-0.83) | 1.0  (1-1) |
| DSC-nCBF median | 3.81 | 0.48  (0.27-0.69) | 0.21  (0.05-0.5) | 1.0  (1-1) | 2.28 | 0.53  (0.2-0.8) | 0.14  (0-0.67) | 1.0  (1-1) |
| DSC-nCBF 95^th^ percentile | 18.04 | 0.43  (0.23-0.66) | 0.14  (0-0.46) | 1.0  (1-1) | 3.36 | 0.44  (0.18-0.73) | 0.8  (0.41-1) | 0.25  (0-0.75) |
| DSC-nCBF IQR | 7.40 | 0.45  (0.23-0.69) | 0.14  (0-0.44) | 1.0  (1-1) | 3.17 | 0.41  (0.15-0.71) | 0.2  (0-0.56) | 1.0  (1-1) |

**Supplementary Table 10**. *Molecular markers prediction performance of univariate DSC-nCBF models.*

|  |  | ASL-nCBF vs DSC-nCBF | | ASL-nCBF vs DSC-nCBV | |
| --- | --- | --- | --- | --- | --- |
| ROI | Histogram parameter | r_s_ | Corrected p-value | r_s_ | Corrected p-value |
| Tumor | 5^th^ percentile | 0.28 | 0.049 | 0.26 | 0.047 |
|  | Median | 0.67 | <0.001 | 0.65 | <0.001 |
|  | 95^th^ percentile | 0.68 | <0.001 | 0.78 | <0.001 |
|  | IQR | 0.64 | <0.001 | 0.74 | <0.001 |
| Edema | 5^th^ percentile | 0.18 | 0.41 | 0.12 | 0.410 |
|  | Median | 0.61 | <0.001 | 0.63 | <0.001 |
|  | 95^th^ percentile | 0.34 | 0.045 | 0.37 | 0.045 |
|  | IQR | 0.36 | 0.049 | 0.45 | 0.005 |

**Supplementary Table 11***. Results of the correlation analysis between ASL and DSC perfusion parameters in tumor and edema.*
